# Soluble CD146 in Heart Failure: Pathophysiological Role and Diagnostic Potential

**DOI:** 10.3390/biomedicines13061370

**Published:** 2025-06-03

**Authors:** Daniela Mocan, Radu Jipa, Daniel Alexandru Jipa, Radu Ioan Lala, Maria Puschita, Florin-Claudiu Rasinar, Diana-Federica Balta, Iulia-Silvia Groza, Amelia Uzum

**Affiliations:** 1 Multidisciplinary Doctoral School, Vasile Goldis Western University of Arad, 310025 Arad, Romania; mocandaniela3@gmail.com (D.M.); radu_lala@yahoo.com (R.I.L.); mpuschita.mp@gmail.com (M.P.); amelia.uzum@yahoo.com (A.U.); 2 Research Center of the Institute of Cardiovascular Diseases Timisoara, 300310 Timisoara, Romania; 3 Department VII, Internal Medicine II, Discipline of Cardiology, University of Medicine and Pharmacy “Victor Babes” Timisoara, E. Murgu Square, Nr. 2, 300041 Timisoara, Romania; 4 Institute of Cardiovascular Diseases of Timisoara, 300310 Timisoara, Romania; 5 Department of “Life Sciences”, Faculty of Medicine, Vasile Goldis Western University of Arad, Romania 86, Liviu Rebreanu Street, 310048 Arad, Romania; 6 Arad County Clinical Emergency Hospital, 310037 Arad, Romania; 7 Doctoral School, Victor Babes University of Timisoara, 300041 Timisoara, Romania; 8 Victor Babes Clinical Hospital for Infectious Diseases and Pneumology of Timisoara, 300041 Timisoara, Romania

**Keywords:** sCD146, heart failure, endothelial dysfunction, congestion, biomarkers, MCAM, heart failure

## Abstract

Heart failure (HF) remains a major global health challenge, driven by multifactorial pathophysiological processes, such as systemic congestion, endothelial dysfunction, and inflammation. While natriuretic peptides are well-established biomarkers for diagnosing and monitoring HF, they do not fully capture the complexity of vascular involvement. CD146, also known as melanoma cell adhesion molecule (MCAM), is a transmembrane glycoprotein primarily expressed on endothelial cells and involved in cell adhesion, vascular permeability, and angiogenesis. Its soluble form (sCD146), released in response to multiple pathophysiological stimuli, including venous and arterial endothelial stretch, oxidative stress, and inflammatory cytokine activation, has emerged as a promising biomarker reflecting both hemodynamic congestion and systemic endothelial stress. This review synthesizes current knowledge on the structure, regulation, and release mechanisms of CD146 and explores its clinical utility in HF. Elevated sCD146 levels have been associated with echocardiographic and radiological indicators of congestion, as well as with adverse outcomes. While promising, its application is limited by variability, lack of standardization, and confounding elevations in non-cardiac conditions, including malignancy.

## 1. Introduction

Heart failure (HF) remains a leading cause of global morbidity and mortality, characterized by complex pathophysiological mechanisms, including impaired cardiac output, neurohormonal activation, systemic congestion, and endothelial dysfunction [1,2,3]. Despite advances in pharmacological and device-based therapies, HF continues to present substantial diagnostic and therapeutic challenges, particularly due to the heterogeneous nature of the syndrome and its overlapping clinical presentations [4].

One of the most significant contributors to HF progression and symptom burden is hemodynamic congestion. Congestion, resulting from elevated intracardiac filling pressures and impaired fluid redistribution, manifests as pulmonary and peripheral edema, ascites, jugular venous distention, and hepatic congestion [5,6,7,8,9,10]. Its presence is not only associated with acute decompensation but is also a major predictor of rehospitalization and adverse outcomes in both acute and chronic HF [1,2,5]. However, traditional clinical assessment of congestion is often subjective and imprecise, highlighting the critical need for more reliable and dynamic biomarkers [4].

In this context, endothelial dysfunction has emerged as a pivotal link between hemodynamic stress and clinical deterioration in HF [11,12,13,14]. The vascular endothelium plays a central role in maintaining vascular tone, permeability, and immune regulation. Disruption of endothelial homeostasis leads to increased vascular permeability, inflammation, and tissue edema, hallmarks of advanced HF [14,15]. Biomarkers that capture this vascular component could significantly enhance our ability to diagnose, stratify, and monitor patients with HF more effectively [1,16,17].

CD146, also known as the melanoma cell adhesion molecule (MCAM), is a transmembrane glycoprotein predominantly expressed on endothelial cells, where it functions in cell adhesion, angiogenesis, and the regulation of vascular permeability [1,18,19]. Its soluble form (sCD146), released into the bloodstream under conditions of endothelial stress, has garnered attention as a potential biomarker of systemic congestion and endothelial injury in HF [1,20].

## 2. CD146: Structure, Expression, and Regulation

CD146 was originally identified in 1987 on the surface of melanoma cells. Since then, its physiological relevance has been substantially redefined, with emerging roles in vascular biology, immune modulation, and tissue remodeling. Now considered a critical adhesion molecule of the endothelial junction, CD146 is deeply involved in maintaining vascular integrity, regulating immune cell trafficking, and responding to environmental stressors such as inflammation and mechanical stretch [16,20,21].

### 2.1. Molecular Structure

Encoded on chromosome 11q23.3 in humans, the CD146 gene spans 16 exons and encodes a 113-kDa transmembrane glycoprotein belonging to the immunoglobulin superfamily, involved in cell adhesion and endothelial signaling [22,23]. The mature CD146 protein comprises an N-terminal signal peptide, a large extracellular domain with five immunoglobulin (Ig)-like domains, eight predicted N-glycosylation sites, a 24-amino-acid hydrophobic transmembrane region, and a short cytoplasmic tail that mediates intracellular signaling. These domains work synergistically to mediate cell–cell adhesion, endothelial cohesion, and downstream signaling cascades involved in cell proliferation, migration, and vascular remodeling [22,24].

The Ig-like domains within the extracellular region of CD146 facilitate both homophilic CD146–CD146 interactions and heterophilic binding with other adhesion molecules, thereby contributing to endothelial cell–cell cohesion and vascular barrier integrity. Glycosylation motifs support proper protein folding, receptor–ligand interactions, and membrane localization, while the cytoplasmic tail engages intracellular signaling cascades that regulate endothelial responses to inflammatory stimuli and vascular injury [24].

### 2.2. Expression Profile

CD146 is predominantly expressed on vascular endothelial cells, particularly at intercellular junctions in both arteries and veins, where it supports endothelial barrier integrity and regulates permeability [1,16,25]. Beyond the endothelium, CD146 is also expressed in vascular smooth muscle cells, where it contributes to vessel remodeling, and in pericytes, which support microvascular stability [26,27,28]. In addition to its vascular localization, CD146 expression has been detected in a variety of extra-vascular tissues, including mesenchymal stromal cells within the bone marrow, trophoblasts in the placenta, and subsets of immune cells, such as T cells, B cells, and natural killer (NK) cells. This broad expression profile highlights the multifunctional nature of CD146, encompassing roles in vascular biology, immune surveillance, and tissue regeneration [23,29].

### 2.3. Isoforms of CD146

Three major isoforms of CD146 have been described: the long form (lgCD146), the short form (shCD146), and the sCD146 [23]. The lgCD146 isoform is localized primarily at the intercellular junctions of endothelial cells and is critical for maintaining cell–cell adhesion and the integrity of the endothelial barrier. In contrast, shCD146 is localized apically on endothelial surfaces and is implicated in dynamic processes such as endothelial proliferation, migration, and wound healing [23].

The third isoform, sCD146, is of particular interest in clinical cardiology [1,20,30,31]. sCD146 is generated through proteolytic cleavage of its membrane-bound form, predominantly mediated by matrix metalloproteinases (MMPs), especially MMP-2 and MMP-9. This shedding process is typically induced by pro-inflammatory cytokines or mechanical endothelial stretch, leading to elevated circulating levels of sCD146 in various pathological conditions, including HF, systemic inflammation, and certain malignancies [23,32]. While sCD146 levels in healthy individuals range between 200 and 400 ng/mL, this reference interval is not yet universally standardized and may be influenced by population demographics, assay specificity, and clinical status [23,33].

### 2.4. Regulation of CD146 Expression and Shedding

CD146 expression and its conversion to a soluble form are regulated by a complex interplay of transcriptional and post-translational mechanisms. Pro-inflammatory cytokines, such as tumor necrosis factor-alpha (TNF-α), interleukin-1β (IL-1β), interleukin-6 (IL-6), and transforming growth factor-beta (TGF-β), are potent inducers of CD146 gene transcription and promote the activation of metalloproteinases that mediate ectodomain shedding [23]. Oxidative stress, primarily mediated by reactive oxygen species (ROS), contributes to the destabilization of endothelial junctions and promotes the proteolytic shedding of CD146, thereby increasing circulating sCD146 levels in conditions characterized by endothelial dysfunction [19].

**Figure 1:**
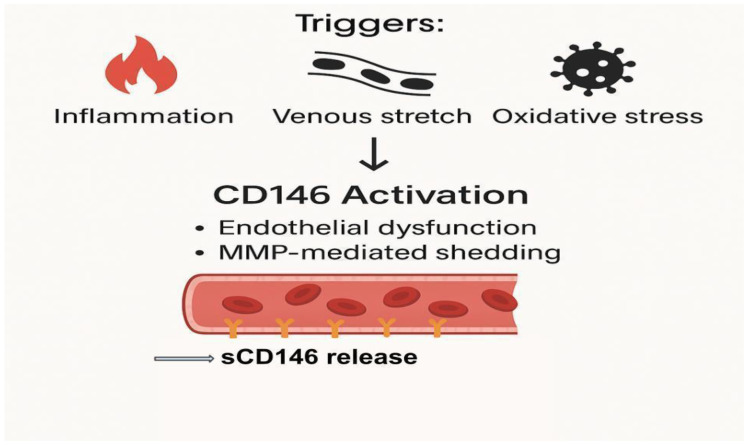
Pathophysiological cascade leading to sCD146 release in heart failure. Inflammation, venous stretch, and oxidative stress act as upstream triggers that induce CD146 activation. This activation is associated with endothelial dysfunction and matrix metalloproteinase (MMP)-mediated shedding of membrane-bound CD146. The result is increased release of sCD146 into the circulation, serving as a potential biomarker of vascular stress and systemic congestion. There is no copyright issue.

In the context of HF, elevated intracardiac and venous pressures result in a mechanical stretch of endothelial cells, a powerful stimulus for CD146 shedding [23]. Additional factors such as hypoxia, ischemia–reperfusion injury, and oxidized low-density lipoprotein (oxLDL) may synergistically enhance both transcriptional upregulation and post-translational modifications of CD146. Collectively, these regulatory mechanisms underscore the dynamic sensitivity of CD146 expression to diverse pathological stimuli, reinforcing the utility of circulating sCD146 as a biomarker of vascular stress and systemic congestion, as illustrated in Figure 1 [19,32].

## 3. Pathophysiological Role of CD146 in Heart Failure: Mechanisms and Vascular Implications

HF is not solely a disorder of impaired myocardial function but also a disease of profound vascular involvement. Central to this vascular dysfunction is the activation and shedding of endothelial markers, among which CD146, particularly its sCD146, has emerged as both a consequence and indicator of systemic congestion and endothelial barrier disruption [16,20,30]. The release of sCD146 is governed by a complex interplay of hemodynamic, inflammatory, oxidative, and hypoxic stimuli that reflect the severity of vascular involvement in HF. Beyond being a passive biomarker, CD146 may also actively contribute to the pathological cascade that perpetuates vascular permeability and congestion [20,21,23].

### 3.1. Hemodynamic Stress and Endothelial Activation

Elevated central venous and intracardiac pressures are hallmark features of both acute and chronic HF [1,20,21,34]. Hemodynamic overload imposes increased mechanical tension on the vascular endothelium, particularly within the highly compliant venous system. Sustained intravascular pressure disrupts endothelial junctional integrity, leading to heightened vascular permeability and facilitating plasma extravasation into the interstitial space. Clinically, this process manifests as peripheral edema, ascites, or pleural effusion, hallmarks of systemic and pulmonary congestion in advanced HF [1,4].

CD146 is constitutively expressed at the intercellular junctions of endothelial cells, where it contributes to vascular cohesion and barrier integrity. In response to pathological hemodynamic stress, such as venous stretch or elevated arterial afterload, mechanical forces activate matrix metalloproteinases, particularly MMP-2 and MMP-9, which cleave membrane-bound CD146. This proteolytic event releases its extracellular domain into the circulation as sCD146, reflecting endothelial perturbation and vascular stress. This release serves as a quantitative marker of endothelial stress, correlating with the degree of vascular strain and systemic congestion [18,23,25].

### 3.2. Inflammatory Cytokines and Oxidative Stress

Inflammation plays a pivotal role in both the initiation and perpetuation of HF pathophysiology. Circulating cytokines such as TNF-α, IL-1β, IL-6, and TGF-β not only contribute to myocardial dysfunction but also trigger endothelial activation, enhance CD146 gene expression, and promote its shedding [7,19,25].

Moreover, the oxidative stress environment in HF, marked by an imbalance between reactive oxygen species (ROS) and antioxidant defenses, directly impairs endothelial integrity and further stimulates MMP activity, potentiating sCD146 release. These inflammatory and oxidative stimuli interact synergistically with mechanical stressors to amplify endothelial injury, linking systemic inflammation to the vascular features of HF [16,19,23,27].

### 3.3. CD146 and Endothelial Dysfunction: Amplifying Congestion

Once released, sCD146 not only reflects endothelial stress but may also contribute to its progression. sCD146 can exert paracrine and autocrine effects, promoting endothelial hyperpermeability, enhancing leukocyte transmigration across the endothelium, and amplifying local inflammatory responses. These actions further compromise vascular integrity and contribute to the progression of inflammatory and cardiovascular disorders [23,32,33]. Specifically, sCD146 interacts with integrins such as αvβ1 on endothelial cells, leading to cytoskeletal remodeling and disruption of tight and adherens junctions. These changes result in endothelial hyperpermeability, facilitating plasma leakage into the interstitial space and contributing directly to tissue congestion.

Moreover, sCD146 enhances leukocyte adhesion and transmigration across the endothelium by upregulating chemokines like IL-8 and increasing the expression of adhesion molecules. This process not only amplifies local inflammatory responses, but also leads to the accumulation of immune cells within the vascular wall and surrounding tissues, further increasing vascular permeability and exacerbating fluid extravasation. These inflammatory and permeability changes compromise vascular integrity, disrupt fluid homeostasis, and contribute to the persistence of both intravascular and extravascular volume overload [23,26,32,33].

Thus, a self-sustaining feedback loop emerges: hemodynamic stress and inflammation promote the shedding of CD146, increasing sCD146 levels, which then worsen endothelial dysfunction, promote vascular leak, and perpetuate systemic and organ congestion. Elevated sCD146 not only marks this pathophysiological state but also actively drives its progression, making it a critical mediator and potential therapeutic target in congestive HF [23,32,33].

## 4. Clinical Relevance and Diagnostic Studies

In the evolving landscape of HF diagnosis, the search for biomarkers that reflect not only myocardial strain, but also vascular dysfunction and congestion, remains a priority [25]. sCD146, a product of endothelial stress and activation, has emerged as a clinically relevant biomarker that complements traditional cardiac-derived indicators, as depicted in Table 1. Unlike natriuretic peptides, which primarily capture myocardial wall stretch, sCD146 reflects endothelial barrier disruption and systemic venous congestion, dimensions central to the pathophysiology of both acute and chronic HF [1,31,34].

### 4.1. Diagnostic Value in Acute and Chronic Heart Failure

Several key studies underscore the diagnostic utility of sCD146 in the setting of acute decompensated heart failure (ADHF). One of the most comprehensive was conducted by Gayat et al., involving a multicenter cohort of patients admitted with ADHF. The study demonstrates that sCD146 concentrations were significantly elevated in patients with clinical and radiographic signs of congestion, independent of left-ventricular ejection fraction (LVEF). Notably, sCD146 showed a diagnostic performance comparable to N-terminal proBNP (NT-proBNP), and the combination of both biomarkers provided enhanced sensitivity and specificity in identifying volume overload [20].

These findings were particularly relevant for patients with HF with preserved ejection fraction (HFpEF), a group in which the diagnosis is often complicated by inconclusive natriuretic peptide levels. Because sCD146 is independent of systolic function and more reflective of vascular and endothelial strain, it has been proposed as a valuable adjunct biomarker, especially in those with ambiguous or non-specific presentations [20].

### 4.2. Associations with Imaging and Hemodynamic Parameters

The credibility of sCD146 as a congestion marker is further supported by its correlation with objective hemodynamic and imaging parameters. In a study by Kuběna et al., elevated sCD146 levels were significantly associated with radiographic signs of pulmonary congestion (e.g., alveolar edema, pleural effusion) in patients with acute coronary syndromes, independent of myocardial injury as assessed by troponin levels. This highlighted the vascular-specific signal of sCD146, distinguishing it from cardiac necrosis markers [31,34,35].

Similarly, Van Aelst et al. observed that sCD146 correlated strongly with echocardiographic markers of systemic congestion, including right atrial enlargement, increased inferior vena cava (IVC) diameter, reduced IVC collapsibility, elevated E/e′ ratio, and increased systolic pulmonary artery pressure (sPAP) [36]. These relationships validate sCD146 as a surrogate marker of right-sided and pulmonary venous congestion, with relevance for bedside clinical evaluation, especially when echocardiographic assessment is limited or inconclusive [36].

Elevated sCD146 levels have been strongly associated with radiographic signs of pulmonary congestion, enlarged right atrial size, inferior vena cava dilation, elevated pulmonary artery pressures, and subclinical congestion, even in patients who appear clinically euvolemic [1,20,34,37]. These correlations confirm the role of sCD146 as a biologically plausible, noninvasive marker of congestion severity that reflects real-time endothelial derangement.

### 4.3. Complementarity to Established Biomarkers

**Figure 2:**
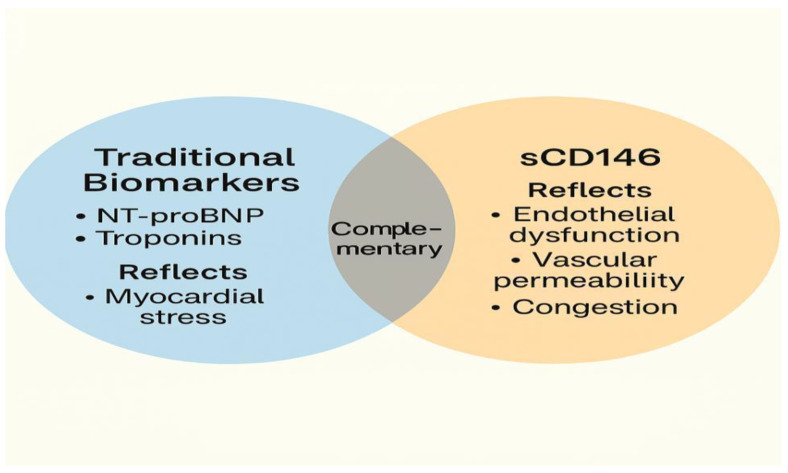
sCD146 as a Biomarker in Heart Failure.

Rather than serving as a replacement for established biomarkers, such as NT-proBNP or troponins, sCD146 should be viewed as complementary, offering distinct yet synergistic information about vascular congestion and endothelial integrity, as depicted in Figure 2 [20,34,36,38]. When combined with NT-proBNP, sCD146 has been shown to improve diagnostic precision in patients with borderline or ambiguous presentations, particularly those with multiple comorbidities or non-specific dyspnea [20].

This diagram illustrates the role of sCD146 as a biomarker for endothelial dysfunction and systemic congestion in HF. The figure shows how sCD146 is released in response to various pathophysiological triggers, such as venous and arterial stretch, oxidative stress, and inflammatory cytokine activation. It highlights the cascade of events leading to endothelial cell activation, proteolytic shedding of sCD146, and its subsequent elevation in circulation. The figure also emphasizes the clinical relevance of sCD146 in diagnosing and monitoring congestion and endothelial integrity, correlating with traditional biomarkers like NT-proBNP and contributing to diagnostic precision in HF management.

## 5. CD146 and Traditional Biomarkers in Heart Failure

### 5.1. Comparative Analysis: CD146 vs. Traditional Biomarkers in Heart Failure

HF is a complex and heterogeneous syndrome characterized by a range of overlapping pathophysiological processes, myocardial stress, systemic congestion, neurohormonal activation, inflammation, and endothelial dysfunction [6,7,35,37,39]. No single biomarker can comprehensively reflect all these components. Therefore, evaluating how sCD146 compares with and complements established HF biomarkers is essential to appreciating its unique and additive value in clinical practice. Table 2 presents a comparative analysis between biomarkers.

Among the traditional biomarkers, natriuretic peptides, namely B-type natriuretic peptide (BNP) and NT-proBNP, remain the cornerstone of HF diagnosis and monitoring. Unlike natriuretic peptides (e.g., NT-proBNP), which are secreted by cardiac myocytes in response to chamber stretch, sCD146 reflects vascular and endothelial components of HF pathology [1,20,40]. This distinction is critical, especially in HFpEF, where myocardial biomarkers may be misleadingly normal, elderly, or obese patients, in whom NT-proBNP levels may be attenuated, post-treatment or residual congestion, where cardiac markers normalize faster than vascular integrity [41]. Cardiac troponins, on the other hand, are highly specific markers of myocardial injury. Their elevation reflects acute or chronic myocardial cell damage and plays a vital role in distinguishing HF with concomitant ischemia or myocardial infarction. Nevertheless, they do not convey information regarding volume status, congestion, or endothelial function [42,43,44].

Emerging biomarkers, including galectin-3, soluble ST2, and MR-proADM, have gained attention for their ability to reflect fibrosis, inflammation, or neurohormonal dysregulation, expanding the biomarker landscape. However, they also face challenges regarding specificity and clinical implementation [1,16,44,45,46,47,48,49].

CD146 is fundamentally different in origin and scope. Unlike NT-proBNP or troponins, which are cardiomyocyte-derived, CD146 is endothelial in origin, reflecting vascular stress, junctional disruption, and permeability alterations. This pathophysiological lens allows sCD146 to offer unique diagnostic and prognostic insights, especially in contexts where endothelial dysfunction plays a central role, such as HF with preserved ejection fraction (HFpEF), systemic congestion, or comorbid inflammatory states [29,31,33,34]. While traditional and emerging biomarkers reflect key components of HF pathophysiology, recent attention has turned to endothelial biomarkers for their potential to enhance short-term prognostic assessment, particularly in acute and hospitalized HF contexts. Among these, syndecan-1, endocan, sCD146, and Vascular Cell Adhesion Molecule 1 (VCAM-1) are gaining prominence for their roles in endothelial injury, inflammation, and congestion. Their ability to capture processes not directly sensed by myocardial or fibrotic markers may provide critical insight into patient trajectories in acute settings [1,20,33]. Syndecan-1, a marker of endothelial glycocalyx degradation, has shown strong associations with adverse outcomes in acute HF. Elevated levels are predictive of 6-month mortality and correlate with systemic inflammation and acute kidney injury [50]. Syndecan-1 may therefore serve as an early warning signal for identifying high-risk patients [51].

Endocan, secreted by activated endothelial cells, rises sharply in patients with cardiogenic shock or severe ADHF. It correlates with disease severity scores (e.g., APACHE II) and natriuretic peptides, and persistent elevation during hospitalization may indicate ongoing endothelitis [52,53]. VCAM-1, associated with cytokine-induced endothelial inflammation, is elevated during acute decompensation and may reflect a systemic inflammatory state. Elevated VCAM-1 levels have been independently associated with adverse long-term outcomes in HF patients [54,55]. Among endothelial biomarkers in acute HF, syndecan-1 appears to be the strongest independent marker of early mortality and multi-organ dysfunction [56]. Endocan and sCD146 provide complementary information—endocan highlighting inflammatory endothelial activation and sCD146 reflecting systemic and pulmonary venous congestion [20,33].

### 5.2. Diagnostic Utility of Integrated Biomarker Panels in Heart Failure

HF involves diverse and overlapping mechanisms, myocardial stretch, inflammation, fibrosis, congestion, and endothelial dysfunction that no single biomarker can fully capture. Panels combining NT-proBNP, soluble ST2 (sST2), and sCD146 show strong potential to enhance diagnostic accuracy by targeting complementary pathophysiological domains: cardiac strain, fibrotic stress, and vascular congestion [20,34,46]. Empirical studies support the synergistic value of this approach, demonstrating that sCD146 performed comparably to NT-proBNP in diagnosing acute decompensated HF (ADHF). Importantly, combining sCD146 with NT-proBNP significantly improved diagnostic precision, particularly in patients with intermediate (“gray zone”) natriuretic peptide levels [20].

In HFpEF, diagnosis remains particularly challenging. In such cases, adjunct markers can reveal pathophysiological changes not reflected by NT-proBNP. For instance, sCD146 was shown by Juknevičienė et al. to be elevated in HFpEF patients with clinical congestion even when NT-proBNP was inconclusive, highlighting its utility in detecting vascular overload [34].

Similarly, sST2 captures the inflammatory and fibrotic component of HF and is unaffected by confounders such as age, obesity, or renal impairment. Elevated sST2 in these patients is associated with worse outcomes, and clinical studies show that adding sST2 to NT-proBNP improves diagnostic confidence and risk stratification [46]. ST2 is now included in HF guidelines with a Class IIb recommendation for selected diagnostic and prognostic scenarios [46].

Further refinements to diagnostic panels may come from incorporating additional endothelial markers. Syndecan-1, a marker of glycocalyx degradation, is elevated in acute HF and correlates with organ dysfunction and early mortality [55]. Its addition to NT-proBNP or troponin-based models enhances prognostic accuracy. Endocan, secreted during endothelial activation, reflects pulmonary hypertension and vascular congestion [51,56]; VCAM-1 is associated with poor long-term outcomes and reflects systemic endothelial activation [52,54]. Taken together, panels combining cardiac markers (NT-proBNP, troponins), fibrotic/inflammatory markers (sST2, galectin-3), and vascular markers (sCD146, syndecan-1, endocan, VCAM-1) offer a comprehensive framework for precision diagnostics in HF. They are particularly useful in diagnostically complex cases, such as HFpEF, obesity, chronic kidney disease, or atypical presentations—where conventional markers may fall short. Integrating these biomarkers into routine diagnostic pathways may significantly improve early detection, individualized treatment, and outcome prediction in both acute and chronic HF [20,34,45,46].

## 6. Limitations and Confounding Conditions

Despite its growing recognition as a biomarker of endothelial dysfunction and vascular congestion, sCD146 (sCD146) remains an investigational tool in managing HF. Several important limitations must be acknowledged when considering its application in research or practice, particularly in the absence of large-scale prospective validation studies [57].

One of the most notable challenges lies in the lack of standardization across available assays for sCD146. Differences in analytical methods, sample preparation, and calibration procedures may lead to inconsistent results between laboratories, complicating the establishment of universal reference ranges or diagnostic thresholds. Moreover, the current literature reflects a heterogeneous mix of study populations, assay types, and clinical endpoints, further limiting the generalizability of existing findings [58].

In addition to methodological concerns, biological variability introduces the potential for misinterpretation. sCD146 levels may be influenced by age, sex, renal function, and systemic inflammatory conditions, factors commonly encountered in HF populations. In particular, non-cardiac elevations have been reported in malignancies, autoimmune diseases, and infectious states. CD146 was originally described as a melanoma cell adhesion molecule and is overexpressed in a range of solid tumors, including breast, prostate, and hepatocellular carcinoma. In such contexts, elevated sCD146 may reflect oncological or immunological processes rather than cardiovascular pathology, thus reducing its specificity as a congestion biomarker [37,45].

Furthermore, the pathophysiological mechanisms underlying sCD146 elevation are still incompletely understood. While mechanical stretch, inflammation, and oxidative stress are known triggers of CD146 shedding, the temporal dynamics and interactions between these stimuli remain to be fully clarified. This gap in mechanistic understanding limits the clinical interpretability of sCD146 in borderline or multifactorial cases, particularly in polymorbid patients with overlapping disease processes. sCD146 has demonstrated correlations with markers of congestion and prognosis in observational studies; it has yet to be tested in prospective biomarker-guided interventional trials [16,59,60]. Its role in therapeutic decision making, patient stratification, or treatment response remains speculative at this stage.

### sCD146 and Renal Dysfunction

Renal dysfunction is a prevalent and prognostically significant comorbidity in HF (HF), affecting up to 50% of patients, especially those with preserved ejection fraction or advanced disease. Notably, kidney dysfunction not only reflects disease severity but also actively influences vascular biology and biomarker behavior, including that of sCD146 [1,2,61].

While sCD146 is primarily released in response to endothelial stress and venous congestion, emerging evidence indicates that impaired renal function can independently elevate sCD146 levels, even in the absence of overt volume overload. In patients with chronic kidney disease (CKD), a reduced glomerular filtration rate (GFR) may hinder renal clearance of sCD146, leading to its accumulation [1,2,18,62].

Beyond impaired elimination, CKD fosters a pro-inflammatory and pro-oxidative vascular milieu that promotes endothelial dysfunction, upregulates CD146 expression, and enhances its shedding via matrix metalloproteinase (MMP-2 and MMP-9) activation [63]. Uremic toxins, such as indoxyl sulfate, and inflammatory cytokines, like TNF-α and IL-6, have been shown to stimulate CD146 release from endothelial cells [64].

These mechanisms result in chronically elevated sCD146 concentrations in patients with moderate-to-severe CKD, independent of HF congestion status. This presents a critical interpretive challenge: in patients with coexisting HF and renal dysfunction—a common and high-risk phenotype—elevated sCD146 may reflect a combination of hemodynamic congestion, endothelial inflammation, and impaired clearance. Consequently, reliance on unadjusted sCD146 levels could lead to overestimation of volume status or endothelial stress in this subgroup [65,66,67].

## 7. Therapeutic Prospects of Targeting CD146 in Heart Failure

While sCD146 is primarily studied as a biomarker, emerging research highlights its active contribution to HF pathophysiology, making it a potential therapeutic target. Under inflammatory or hemodynamic stress, membrane-bound CD146 is cleaved by matrix metalloproteinases (MMP-2 and MMP-9), releasing sCD146 into circulation [33,50,68]. This soluble form promotes leukocyte transmigration, vascular permeability, and endothelial activation, exacerbating congestion and multi-organ dysfunction [22,32]. Meanwhile, loss of membrane CD146 destabilizes endothelial junctions, worsening capillary leak and inflammation [24,50].

Several therapeutic strategies have been proposed to counteract this cascade:

- MMP inhibition aims to preserve junctional CD146 by preventing its proteolytic cleavage. Although broad-spectrum MMP inhibitors have shown limited clinical success in HF due to side effects, selective MMP blockade could reduce endothelial injury with fewer off-target effects [54,68].
- Neutralization of sCD146 is an alternative approach. The monoclonal antibody M2J-1, developed to specifically bind sCD146 while sparing the membrane-bound form, has shown efficacy in preclinical cancer models, reducing pathological angiogenesis and inflammation without compromising vascular stability [38,69]. This targeted strategy may have relevance in HF, especially in HFpEF, where persistent congestion and endothelial inflammation are key drivers of disease [14,19].
- Direct blockade of endothelial CD146 has also been explored. In neuroinflammation models, the monoclonal antibody AA98 reduced leukocyte extravasation and tissue injury by disrupting CD146-mediated immune cell adhesion [36,70]. Translating this concept to HF could help modulate chronic inflammatory infiltration in the myocardium and peripheral organs, though careful dosing would be needed to avoid impairing normal immune function.

These mechanisms are particularly promising in HFpEF, which is characterized by microvascular inflammation, endothelial dysfunction, and diastolic stiffening [15,19]. In this phenotype, CD146-targeted therapy could mitigate disease progression by restoring barrier function and reducing cytokine-induced vascular permeability. In HFrEF, while myocyte loss and neurohormonal activation dominate early disease, chronic systemic congestion and immune activation play increasing roles. CD146 inhibition may help alleviate residual organ congestion and vascular injury, improving quality of life and functional status [14,50].

Although no HF therapies currently target CD146 directly, guideline-directed medications, including ARNI, β-blockers, MRAs, and SGLT2 inhibitors, partially overlap [48,71]. For instance, MRAs reduce MMP activity and oxidative stress and SGLT2 inhibitors have been shown to improve endothelial function and lower inflammatory signaling [46,72]. These effects may indirectly modulate CD146 shedding, though the pathway remains largely unaddressed by existing treatments.

Notably, recent pooled analyses from DELIVER and EMPEROR-Preserved trials confirmed the efficacy of SGLT2 inhibitors across a broad ejection fraction range, highlighting the clinical relevance of vascular and metabolic mechanisms in HF therapy [1,2,40,73,74,75,76,77]. Adding CD146-modulating strategies to this armamentarium could address persistent endothelial dysfunction and systemic congestion, which are only partially controlled by current regimens.

In conclusion, CD146 is more than a biomarker, it is a mediator of vascular injury and inflammation in HF. Therapeutic strategies targeting MMP-mediated shedding, neutralizing sCD146, or modulating endothelial CD146 function could offer novel, mechanism-based interventions in both HFpEF and HFrEF. These approaches warrant translational studies to evaluate their additive potential alongside established therapies and their ability to improve clinical outcomes in this complex and heterogeneous syndrome.

## 8. Conclusions

CD146, particularly in its soluble form (sCD146), has emerged as a compelling biomarker at the intersection of vascular biology and HF pathophysiology. Its expression on endothelial cells and regulated release in response to mechanical, inflammatory, and oxidative stimuli provide a unique window into the endothelial response to systemic congestion, a central yet often underappreciated feature of HF progression [6,16,59].

Unlike traditional biomarkers, such as natriuretic peptides, which predominantly reflect myocardial stretch and intracardiac pressure, sCD146 captures an endothelial and vascular perspective [26,34,77]. This distinction is especially valuable in diagnostically ambiguous cases, such as those with preserved ejection fraction, obesity, renal dysfunction, or residual subclinical congestion, where conventional markers may fall short [1,16,34].

While the current evidence underscores the diagnostic and prognostic promise of sCD146, its use in clinical practice remains limited by biological variability, assay non-standardization, and a lack of prospective interventional data. Nonetheless, its consistent associations with congestion markers, endothelial dysfunction, and adverse outcomes support further investigation and integration into multi-marker strategies for a more nuanced assessment of HF [17].

Moving forward, validation in large-scale cohorts, mechanistic elucidation, and biomarker-guided therapeutic studies are needed to define the clinical role of CD146. If successfully integrated, CD146 could help personalize HF care by refining diagnosis, enhancing prognostication, and guiding decongestive management, ultimately aligning with the goals of precision medicine in cardiovascular disease [1].

## Figures and Tables

**Table 1:** Clinical Applications of sCD146 in Heart Failure.

Application	Details
Diagnostic Utility	Elevated sCD146 levels are associated with systemic and pulmonary congestion, reflecting endothelial dysfunction and vascular strain. It complements traditional biomarkers like NT-proBNP in diagnosing HF.
Prognostic Value	High sCD146 levels predict adverse outcomes, including rehospitalization, disease progression, and mortality. It provides independent prognostic information, especially in conjunction with other biomarkers.
Monitoring Therapy	Serial measurements of sCD146 can be used to monitor treatment response, especially in decongestive therapy. Reductions in sCD146 levels may indicate effective decongestion and improved endothelial function.
Utility in HF with Preserved Ejection Fraction (HFpEF)	sCD146 is valuable in diagnosing and monitoring HFpEF, where traditional markers like NT-proBNP may be less reliable.
Identification of Subclinical Congestion	Persistent elevation of sCD146 levels may indicate residual congestion even after apparent clinical improvement, identifying patients at risk for relapse.

Abbreviations: sCD146 (soluble CD146), NT-proBNP (N-terminal proBNP), HFpEF (heart failure with preserved ejection fraction).

**Table 2:** Comparative Analysis: CD146 vs. Traditional Biomarkers in Heart Failure.

Biomarker	Source	Primary Signal	Clinical Strengths	Limitations
NT-proBNP	Cardiomyocytes	Myocardial wall stretch	High sensitivity for volume overload; prognosis	Affected by renal function, obesity
Troponin I/T	Cardiomyocytes	Myocyte necrosis	Acute coronary syndrome, myocardial injury	Does not reflect congestion
Galectin-3	Fibroblasts	Fibrosis, inflammation	Risk stratification	Low specificity
sST2	Immune cells, myocardium	Cardiac stress, inflammation	Prognosis	Influenced by comorbidities
sCD146	Endothelial cells	Endothelial dysfunction, congestion	Complements NT-proBNP; reflects vascular strain and central congestion	Elevated in malignancy, inflammatory diseases; limited outcome data
Syndecan-1	Endothelial glycocalyx	Glycocalyx degradation, microvascular damage	Independent predictor of early mortality, reflects systemic endothelial injury	Elevated in other critical illnesses (e.g., sepsis); lacks cardiac specificity
Endocan	Activated endothelial cells	Endothelial inflammation and dysfunction	Elevated in cardiogenic shock; correlates with BNP and disease severity	Prognostic role in HF still emerging; not specific to HF
VCAM-1	Cytokine-activated endothelium	Leukocyte adhesion and inflammation	Reflects cytokine-driven endothelial inflammation; part of the acute HF inflammatory profile	Prognostic power unclear in acute HF; overlap with CRP and other inflammatory markers

Abbreviations: NT-proBNP (N-terminal proBNP), sST2 (Soluble Suppression of Tumorigenicity-2), sCD146 (soluble CD146), HF (heart failure), CRP (C-reactive protein).

## Abbreviations

The following abbreviations are used in this manuscript:

HF	Heart failure
NYHA	New York Heart Association
RAAS	Renin–angiotensin–aldosterone system
SNS	Sympathetic nervous system
ADH	Antidiuretic hormone
NO	Antidiuretic hormone
ROS	Reactive oxygen species
CKD	Chronic kidney disease
lgCD146	long form CD146
shCD146	short form CD146
sCD146	soluble form CD146
MMP	Matrix metalloproteinases
